# Dr. John H. Wiggins

**Published:** 1907-05

**Authors:** 


Dr. John H. Wiggins, of Jamestown, N. Y., died at his home in that city April 1, 1907, aged 53 years. He graduated in medicine at the University of Buffalo in 1879.

